# Childhood Obesity and the Future Burden of Adult Cardiometabolic Disease: A Narrative Review

**DOI:** 10.7759/cureus.111102

**Published:** 2026-06-18

**Authors:** Harikrishnan Balakrishna, Arya Ajith, Sreekumar Sasi, Suja M Sukumaran

**Affiliations:** 1 Department of Health Services, Government of Kerala, Thiruvananthapuram, IND; 2 Department of Paediatrics, Lancashire Teaching Hospitals NHS Foundation Trust, Preston, GBR

**Keywords:** cardiometabolic disease, cardiovascular disease, childhood obesity, global burden of disease, life course epidemiology, mendelian randomisation, metabolically healthy obesity, prevention, public health, type 2 diabetes mellitus

## Abstract

Childhood obesity is a growing public health concern with significant long-term implications for cardiometabolic health. Without effective intervention, the global burden of childhood and adolescent obesity is projected to increase substantially in the coming decades.

This study aimed to synthesise current evidence on the epidemiology, pathophysiology, causal mechanisms, longitudinal outcomes, social and commercial determinants, and preventive strategies related to childhood obesity and adult cardiometabolic disease from a public health perspective.

A narrative review was conducted and reported in accordance with the Scale for the Assessment of Narrative Review Articles (SANRA) framework. Literature was identified through searches of PubMed/MEDLINE, Scopus, and Google Scholar from January 2000 to January 31, 2026. A total of 348 records were identified, of which 38 sources were retained for citation. Landmark pre-2000 studies were additionally identified through targeted hand searching.

This review examined the epidemiology, pathophysiology, longitudinal tracking, causal mechanisms, metabolic phenotyping, social and commercial determinants, and preventive strategies related to childhood obesity and adult cardiometabolic disease. Consistent evidence was identified linking childhood obesity to elevated long-term cardiometabolic risk. Both metabolically healthy and metabolically unhealthy obesity phenotypes in childhood are associated with increased cardiometabolic risk in adulthood. Socioeconomic and ethnic inequalities in childhood obesity continue to widen across diverse settings, while emerging evidence highlights a growing dual burden of undernutrition and excess adiposity in transitional populations. Weight normalisation before adulthood appears to substantially reduce future cardiometabolic risk and remains an important clinical objective.

Childhood obesity is associated with an increased risk of adult cardiometabolic disease through interconnected biological, social, environmental, and commercial pathways. Clinical practice should incorporate systematic cardiometabolic risk assessment in children with obesity regardless of current metabolic status. Future research should prioritise trans-ethnic causal studies, long-term cardiometabolic outcome trials in paediatric populations, and evaluation of structural policy interventions.

## Introduction and background

Childhood obesity has shifted from a concern primarily affecting high-income nations to one of the defining global public health challenges. The Global Burden of Disease Study (GBD) 2021, modelling 204 countries from 1990 to 2021 and projecting to 2050, found that both overweight and obesity increased substantially in every world region [1]. The Non-communicable Diseases Risk Factor Collaboration (NCD-RisC) confirmed 159 million school-aged children living with obesity in 2022, representing 9.3% of boys and 6.9% of girls aged 5-19 years globally [2]. If current trajectories continue, 360 million children and adolescents will be living with obesity by 2050 [1]. A higher-than-optimal body mass index (BMI) caused an estimated 3.7 million deaths from noncommunicable diseases (NCDs) such as cardiovascular diseases, diabetes, etc. [3]. A growing body of longitudinal evidence demonstrates that obese children carry a disproportionate lifetime burden of these diseases [4-6], with atherosclerotic injury beginning in childhood, detectable at autopsy in obese adolescents and by noninvasive vascular assessment [7,8].

Obesity in children is defined using age- and sex-specific BMI percentile thresholds, with the 95th percentile commonly used as the clinical cutoff in epidemiological and clinical contexts [9,10]. The mechanisms linking childhood obesity to adult cardiometabolic disease are multifactorial, encompassing insulin resistance, chronic low-grade inflammation, endothelial dysfunction, oxidative stress, and altered adipokine and gut-derived signalling [9,10]. These observations align with the Developmental Origins of Health and Disease (DOHaD) hypothesis, which proposes that exposures during critical developmental windows programme long-term metabolic vulnerability [11]. Mendelian randomisation has strengthened causal inference by demonstrating that genetically predicted higher childhood BMI independently increases the risk of coronary heart disease, myocardial infarction, heart failure, and atrial fibrillation [12]. Metabolically healthy obesity (MHO) refers to obesity in the absence of concurrent metabolic syndrome components at the time of assessment, while metabolically unhealthy obesity (MUO) denotes obesity with established metabolic abnormalities including dyslipidaemia, hyperglycaemia, or elevated blood pressure; both phenotypes carry substantially elevated long-term cardiometabolic risk compared with metabolically healthy normal-weight peers. Systematic review evidence has challenged the concept of MHO as a safe phenotype, confirming that MHO children carry substantially elevated long-term cardiometabolic risk [13]. This narrative review synthesises the epidemiology, pathophysiology, causal evidence, social and commercial determinants, and preventive strategies related to childhood obesity and adult cardiometabolic disease.

## Review

Methods

This narrative review was conducted and reported in accordance with the Scale for the Assessment of Narrative Review Articles (SANRA) framework [14]. A narrative design was selected because of the thematic breadth of the research question, spanning epidemiology, pathophysiology, genetic epidemiology, social determinants, and prevention across the life course, which precluded a meta-analytic pooling. The structured search reflects SANRA guidance for transparent reporting rather than systematic review methodology; hence, prospective registry registration was not required and not performed. Literature was identified through PubMed/MEDLINE, Scopus, and Google Scholar from January 2000 to January 31, 2026, supplemented by reference list screening and targeted hand searches for landmark pre-2000 studies. Search terms included combinations of childhood obesity, cardiometabolic disease, type 2 diabetes mellitus (T2DM), cardiovascular disease, life course epidemiology, Mendelian randomisation, BMI trajectory, MHO, socioeconomic determinants, and related terms.

A total of 348 records were identified, and 38 sources were retained. Landmark pre-2000 studies were additionally identified through a targeted hand search of the reference lists. Priority was given to systematic reviews, meta-analyses, longitudinal cohort studies, Mendelian randomisation analyses, clinical guidelines, and major public health reports, with preference for prospective cohort designs, large sample sizes, and extended follow-up periods where available. The final evidence base comprised systematic reviews and meta-analyses (n≈10), prospective cohort studies (n≈12), Mendelian randomisation analyses, clinical guidelines, randomised controlled trials (RCTs), and major public health reports. For intervention evidence, preference was given to Cochrane reviews and large RCTs. For genetic epidemiology, two-sample Mendelian randomisation analyses using large genome-wide association study datasets were given preference. Study quality was evaluated contextually within each thematic section, with weight given to sample size, prospective design, follow-up duration, and analytical approach to confounding, consistent with SANRA guidance for narrative synthesis. Where multiple studies addressed the same question, the most recent systematic review or meta-analysis was preferentially cited.

Global epidemiology, socioeconomic inequalities, and burden

GBD 2021 forecasting data identify the 2025-2030 period as a critical window. Without decisive action before 2030, a prevalence spike in low- and middle-income countries (LMICs) will precipitate significant public health challenges in regions with limited health system capacity [1]. While historically the highest prevalence rates were concentrated in North America, Australia, and parts of Europe, the fastest absolute increases are now occurring in South Asia, East Asia, the Middle East, North Africa, and sub-Saharan Africa, regions simultaneously managing residual undernutrition and the emerging dual burden of overnutrition [1,2]. In LMICs undergoing nutritional transition, obesity is initially concentrated in higher-income urban groups before broadening across socioeconomic strata as food environments westernise [15]. Adolescence represents a particularly vulnerable window. Pubertal hormonal changes, increasing autonomy over dietary choices, and heightened susceptibility to digital food marketing cumulate to create conditions in which weight gain is common and preventive intervention is often insufficient [16]. 

A consistent finding across high-income countries is that aggregate prevalence figures conceal deepening socioeconomic and ethnic inequalities. In the United Kingdom, overall childhood obesity prevalence appeared to plateau after 2004, but this concealed widening inequalities driven by increasing prevalence among socioeconomically disadvantaged children. The gap between the least and most educationally advantaged households widened from 1.1% to 13.2% between 1995 and 2019 [17]. Similar gradients have been documented in the United States, Australia, and across European Union member states [1,2]. These patterns reflect intergenerational cycles of poverty, food insecurity, and neighbourhood-level food environment inequalities. In LMICs, the burden is rapidly growing. India's Comprehensive National Nutrition Survey (CNNS) found an overweight and obesity prevalence of 4.8% among adolescents aged 10-19 years [18]. Indian childhood obesity (ages 5-9 years) is separately projected to contribute 11% of the global burden by 2030 [18,19]. A two-decade Indian meta-analysis confirmed a consistent socioeconomic gradient with urban and higher-income children at greatest risk [19]. In Brazil, childhood obesity prevalence and associated healthcare costs increased significantly from 2013 to 2022 [20]. These country-specific patterns confirm that while the biological pathways to cardiometabolic disease are universal, the structural drivers differ substantially between settings. Recent global analyses indicate that many African countries are undergoing an obesity transition characterised by rising obesity prevalence alongside persistent undernutrition. While the burden varies across regions, including North Africa and sub-Saharan Africa, the coexistence of undernutrition and excess adiposity presents a growing public health challenge. This evolving double burden highlights the need for context-specific prevention strategies and greater longitudinal research examining the long-term cardiometabolic consequences of childhood obesity in African populations [1,2].

Pathophysiological mechanisms linking childhood obesity to cardiometabolic risk

Childhood obesity initiates a cascade of interconnected metabolic derangements beginning well before clinical disease becomes apparent [9,10]. Excess visceral adiposity drives insulin resistance through the elevated secretion of free fatty acids and pro-inflammatory adipokines, including tumour necrosis factor alpha (TNF-α) and interleukin 6 (IL-6), while adiponectin is paradoxically reduced, impairing insulin signalling in the skeletal muscle, liver, and adipose tissue and driving compensatory hyper-insulinaemia and progressive beta-cell exhaustion [21]. An atherogenic lipid profile comprising elevated triglycerides, elevated low-density lipoprotein (LDL) particles, and reduced high-density lipoprotein (HDL) cholesterol compounds vascular injury and is directly correlated with coronary arterial atherosclerosis [7]. Chronic low-grade systemic inflammation promotes endothelial dysfunction and atherogenesis [10]. Hypertension develops through renin-angiotensin-aldosterone system (RAAS) activation, increased cardiac output, heightened sympathetic tone, and structural renal changes, with blood pressure tracking significantly from childhood into adulthood [21]. Hepatic steatosis, now reclassified as metabolic dysfunction-associated steatotic liver disease (MASLD), previously non-alcoholic fatty liver disease (NAFLD) [22], is recognised as an independent marker of systemic cardiometabolic risk [9,21]. Mendelian randomisation confirms that HDL cholesterol, triglycerides, hypertension, and T2DM together mediate the causal pathway from childhood adiposity to adult coronary heart disease, myocardial infarction, heart failure, and atrial fibrillation [12]. Metabolic syndrome affects an estimated 30-50% of adolescents with obesity, substantially amplifying risk [21]. Consistent with the DOHaD framework, obesity during critical developmental windows induces epigenetic modifications, including DNA methylation and histone acetylation, that alter gene expression governing adipogenesis and insulin sensitivity, potentially perpetuating cardiometabolic vulnerability across generations [9,11].

**Figure 1:**
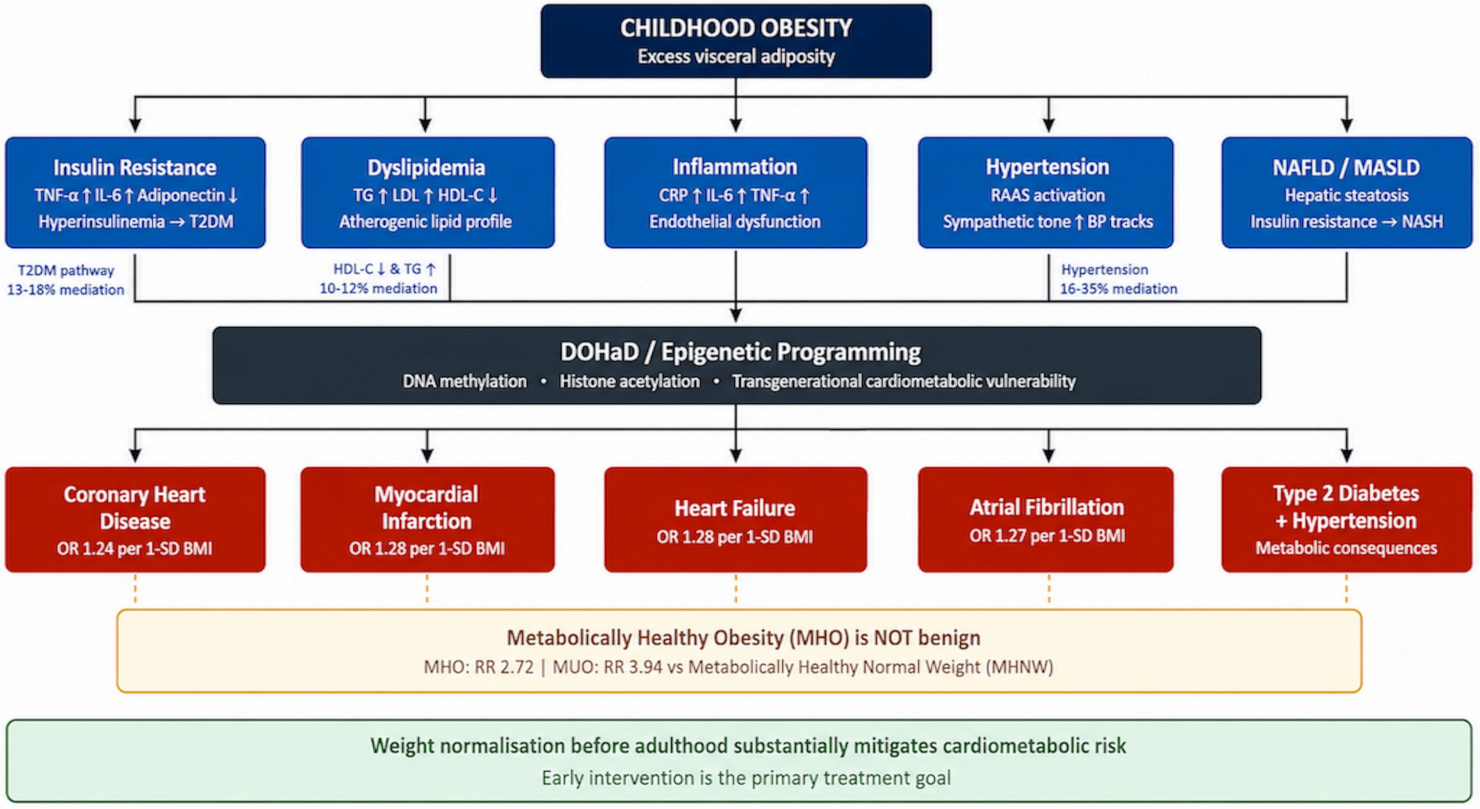
Pathophysiological pathways from childhood obesity to adult cardiometabolic disease showing grouped pathways TNF-α: tumour necrosis factor alpha; IL-6: interleukin 6; T2DM: type 2 diabetes mellitus; TG: triglycerides; LDL: low-density lipoprotein; HDL-C: high-density lipoprotein cholesterol; CRP: C-reactive protein; RAAS: renin-angiotensin-aldosterone system; BP: blood pressure; NAFLD: non-alcoholic fatty liver disease; MASLD: metabolic dysfunction-associated steatotic liver disease; NASH: non-alcoholic steatohepatitis; DOHaD: Developmental Origins of Health and Disease; BMI: body mass index; MHO: metabolically healthy obesity; MUO: metabolically unhealthy obesity Figure created by the authors using Google Slides (Google LLC, Mountain View, California, United States)

Two additional pathophysiological domains are also important. First, childhood obesity fundamentally disrupts hypothalamic appetite regulation. Chronically elevated leptin paradoxically fails to suppress appetite due to receptor desensitisation (leptin resistance), while dysregulated ghrelin perpetuates caloric overconsumption [16]. These disruptions converge on pro-opiomelanocortin (POMC) and agouti-related peptide (AgRP)/neuropeptide Y (NPY) neurons in the arcuate nucleus of the hypothalamus. Glucagon-like peptide 1 (GLP-1), produced by intestinal L cells and acting on hypothalamic receptors to promote satiety, is secreted at reduced levels in obesity, providing the biological rationale for GLP-1 receptor agonists as therapeutic agents [16,23]. Second, the gut microbiome is an increasingly recognised pathophysiological contributor. Obese children demonstrate gut microbial dysbiosis characterised by reduced alpha diversity, an altered *Firmicutes*-to-*Bacteroidetes* ratio, and increased pro-inflammatory taxa [24]. Reduced populations of beneficial bacteria impair intestinal barrier integrity, reduce the production of short-chain fatty acids (SCFAs) including butyrate, and activate toll-like receptor 4 signalling via microbially derived lipopolysaccharide, perpetuating the chronic low-grade inflammation that underlies insulin resistance and atherogenesis [24]. The pathophysiological pathways are summarised in Figure 1.

Tracking of childhood obesity into adulthood

The evidence that obesity tracks from childhood into adulthood is robust and consistent across populations. Simmonds et al., in a systematic review and meta-analysis of 15 prospective cohort studies involving 200,777 participants, found that obese children were approximately five times more likely to be obese as adults (pooled RR: 5.21; 95% CI: 4.50-6.02), approximately 55% of obese children become obese adolescents, 80% of obese adolescents remain obese in adulthood, and 70% will be obese beyond age 30 [25]. Kibret et al., examining life course BMI trajectories across 17 cohort studies, demonstrated that a persistently overweight trajectory was associated with a significantly higher risk of hypertension (RR: 2.49; 95% CI: 1.9-3.28) and T2DM (RR: 4.62; 95% CI: 2.36-9.04) compared with a stable normal BMI trajectory [6]. Juonala et al., in a multi-cohort analysis across North America, Finland, and Australia, found that children with obesity who normalised weight by adulthood had cardiovascular risk profiles similar to those who had never been obese [5], establishing weight normalisation before adulthood as the primary clinical goal and providing a compelling evidence base for sustained early intervention.

Adult cardiometabolic outcomes and causal evidence

The strongest evidence linking childhood obesity to adult cardiometabolic disease derives from longitudinal cohort studies, trajectory analyses, and Mendelian randomisation. Table 1 summarises key evidence published up to January 2026. 

**Table 1:** Summary of major longitudinal cohort studies, trajectory meta-analyses, and causal analyses linking childhood obesity to adult cardiometabolic outcomes BMI: body mass index; LDL-C: low-density lipoprotein cholesterol; BP: blood pressure; CVD: cardiovascular disease; T2DM: type 2 diabetes mellitus; MHO: metabolically healthy obesity; MUO: metabolically unhealthy obesity; MHNW: metabolically healthy normal weight; CHD: coronary heart disease; IMT: intima-media thickness; MI: myocardial infarction; HF: heart failure; AF: atrial fibrillation; OR: odds ratio; RR: relative risk; SR: systematic review; MA: meta-analysis; MR: Mendelian randomisation

Study (year)	Country	N	Follow-up/design	Key cardiometabolic finding	Evidence level
Bogalusa Heart Study [7]	USA	>10,000	Up to 38-year cohort + autopsy findings	Atherosclerotic lesions correlated with childhood BMI, LDL-C, and BP at autopsy; atherogenesis confirmed to begin in childhood	Moderate: cohort + autopsy findings
Young Finns Study [8]	Finland	2,229	21-year prospective cohort	Childhood BMI, LDL-C, and systolic BP independently predicted increased carotid IMT in adulthood	Moderate: prospective cohort
Copenhagen School Health Records [4]	Denmark	276,835	CHD events from age 25; 5.1 million person-years	CHD hazard ratios 1.10 (age 7) to 1.24 (age 13) per 1 unit BMI z-score; birth weight adjustment strengthened associations	High: large prospective cohort
Juonala multi-cohort [5]	USA/Finland/Australia	6,328	Mean 23-year prospective cohort	Weight normalisation before adulthood restored CVD risk to levels seen in those never obese	High: multi-cohort prospective
Harvard Growth Study [26]	USA	508	55-year historical cohort	RR 2.3 for CHD mortality and RR 1.8 for all-cause mortality in overweight adolescent males, independent of adult weight	Moderate: historical cohort; survival bias
Israeli military cohort [27]	Israel	2.3 million	Study period from 1967 to 2010; prospective cohort	Dose-dependent association between adolescent BMI and cardiovascular death; elevated risk within the high normal BMI range	High: very large prospective cohort
BMI trajectory SR/MA [6]	International (17 cohorts)	Multiple	SR and MA	Persistently overweight trajectory: RR 2.49 for hypertension and RR 4.62 for T2DM vs. stable normal BMI throughout the life course	High: SR and MA
Mendelian randomisation [12]	European ancestry GWAS data	39,620	Two-sample MR	OR 1.24 CHD, OR 1.28 MI, OR 1.28 HF, and OR 1.27 AF per 1-SD childhood BMI; European ancestry only, trans-ethnic validation required	Very high: MR; ancestry limitation
Childhood obesity phenotypes SR/MA [13]	International	7,270 (3 cohorts) in a total population of 8,446 (4 cohorts)	SR and MA	MHO: RR 2.72 for adult diabetes vs. MHNW; MUO: RR 3.94 vs. MHNW; supports broad preventive efforts	High: SR and MA

The Bogalusa Heart Study provided the earliest autopsy-confirmed evidence that atherogenesis begins in childhood [7]. Baker et al., using 276,835 Danish schoolchildren followed over 5.1 million person-years, demonstrated coronary heart disease hazard ratios rising from 1.10 at age 7 to 1.24 at age 13 per 1 unit BMI z-score increase in boys, with birth weight adjustment strengthening rather than attenuating associations [4]. Must et al., in the 55-year Harvard Growth Study follow-up, found a relative risk of 2.3 for coronary heart disease mortality and a relative risk of 1.8 for all-cause mortality in overweight adolescent males, independent of adult weight [26]. Twig et al., following 2.3 million Israeli adolescents from 1967 to 2010, demonstrated a dose-dependent continuous relationship between adolescent BMI and cardiovascular death, with elevated risk apparent even within the high normal BMI range [27]. Despite their scale and duration, these cohort studies share inherent limitations, including residual confounding from unmeasured lifestyle factors, secular changes in obesity classification, and differential attrition. Consistency of findings across geographically and methodologically diverse cohorts substantially strengthens confidence in the observed associations.

Xiong et al. provided the most comprehensive causal evidence using two-sample Mendelian randomisation, demonstrating that every 1 SD increase in genetically predicted childhood BMI was causally associated with an OR of 1.24 (95% CI: 1.12-1.37) for coronary heart disease, an OR of 1.28 (95% CI: 1.14-1.42) for myocardial infarction, an OR of 1.28 (95% CI: 1.14-1.42) for heart failure, and an OR of 1.27 (95% CI: 1.04-1.49) for atrial fibrillation, mediated by HDL cholesterol, triglycerides, hypertension, and T2DM. While Mendelian randomisation substantially reduces confounding and reverse causation, findings remain subject to important caveats. Horizontal pleiotropy, whereby genetic instruments influence outcomes through pathways independent of adiposity, may bias effect estimates, though sensitivity analyses using the inverse variance‐weighted method found no evidence of significant directional pleiotropy. Genetically predicted lifelong adiposity may not fully replicate heterogeneous environmentally driven obesity trajectories in contemporary populations, as all genome-wide association study datasets were from European-ancestry populations, hence limiting direct generalisation to South Asian, East Asian, and African populations experiencing the fastest growth in childhood obesity burden [12]. Trans-ethnic Mendelian randomisation using ancestrally diverse datasets represents the most urgent methodological priority. Huang et al., in a systematic review and meta-analysis encompassing four cohort studies involving 8,446 participants (with the diabetes-specific meta-analysis drawing on three cohort studies involving 7,270 participants), demonstrated that MHO children had a pooled RR of 2.72 (95% CI: 1.14-6.48) for adult diabetes compared with MHNW peers, while metabolically unhealthy obese children had a pooled RR of 3.94 (95% CI: 2.77-5.60) vs. MHNW [13]. All children with obesity require preventive intervention regardless of metabolic status at assessment.

Social, commercial, and environmental determinants of childhood obesity

Childhood obesity is profoundly shaped by the social, economic, physical, and digital environments in which children develop. Ultraprocessed foods (UPFs), characterised by industrial formulation, high energy density, and low nutritional quality, have been identified as a major global driver [15]. The UK National Diet and Nutrition Survey data found UPFs accounted for 56.8% of dietary energy intake [28], reflecting a broader global trend of dietary westernisation now affecting populations in South Asia, Latin America, and sub-Saharan Africa [15]. The commercial determinants of health represent an under-appreciated but powerful driver of the epidemic. Transnational food corporations, digital advertising platforms, and algorithm-driven social media systems actively shape children's food preferences through targeted marketing, influencer promotion, and engagement-maximising content amplification [29]. The Lancet series on commercial determinants of health demonstrates that the UPF corporations are among the four major industry sectors collectively responsible for a substantial share of preventable global mortality and calls for various governance mechanisms that constrain harmful commercial practices rather than relying on the industry's self-regulation [29]. Adverse childhood experiences, including abuse, neglect, parental mental illness, and household substance misuse, are associated with both childhood obesity and adult cardiometabolic disease [30,31], operating through chronic cortisol elevation, upregulating NPY, and impairing leptin sensitivity, driving preference for energy-dense UPFs [31]. Cumulative multiple disadvantages, including food insecurity, reduced access to safe physical activity spaces, greater digital and commercial media exposure, and household instability, mean that socioeconomically deprived children face a cumulative obesogenic burden that individually focused interventions cannot address without concurrent structural change [1,29].

Prevention strategies and public health interventions

Effective prevention requires multifaceted approaches across individual, family, community, and structural levels. Table 2 summarises evidence-based interventions across the life course.

**Table 2:** Evidence-based interventions for childhood obesity prevention and management across the life course GDM: gestational diabetes mellitus; WHO: World Health Organization; PEN: Package of Essential NCD Interventions; SDIL: Soft Drinks Industry Levy; SSB: sugar-sweetened beverage; RCT: randomised controlled trial; LMICs: low- and middle-income countries; DOHaD: Developmental Origins of Health and Disease; GLP-1: glucagon-like peptide-1 receptor agonist; SR: systematic review; MA: meta-analysis; BMI: body mass index

Level	Intervention approach and examples	Key evidence and findings
Prenatal and early life	WHO antenatal care recommendations: gestational weight management; GDM screening; Baby Friendly Hospital Initiative breastfeeding promotion [32]	Breastfeeding associated with a 13-26% childhood obesity risk reduction. Maternal pre-pregnancy obesity, excess gestational weight gain, and GDM are the strongest prenatal predictors, operating through fetal programming aligned with DOHaD [11]
School-based	WHO Health Promoting Schools framework: multicomponent dietary and physical activity programmes; national school food policies globally [33]	153 RCTs in Cochrane 2019. Significant BMI z-score reductions with combined programmes sustained over at least one academic year, with family involvement and cultural tailoring [33]. Evidence from LMICs growing but sparse [34]
Family-centred and community	WHO PEN: family-based behavioural interventions targeting parenting practices, meal patterns, screen time, and home food environment; community health worker-delivered programmes [35]	Family-based weight management delivers clinically meaningful benefit when sustained over six months [35]. Whole-community approaches have demonstrated sustained population-level reductions in childhood overweight. Community approaches addressing food access and built environment show potential in disadvantaged populations [1,33]
Fiscal and regulatory	SSB taxes (UK SDIL 2018, Mexico 2014, Chile 2016, South Africa 2018); graphic front-of-pack warning labels; restrictions on marketing of energy-dense foods to children globally [36,37]	SR/MA across multiple countries confirms that SSB taxes reduce purchases and dietary sugar [36]. UK SDIL demonstrated equity benefit in lower-income households [37]. Evidence strong for purchasing behaviour and product reformulation; emerging for direct paediatric BMI z-score reduction in isolation
Pharmacological (adjunct)	GLP-1 receptor agonists (semaglutide, liraglutide) as adjuncts to lifestyle intervention in adolescents with severe obesity and established cardiometabolic comorbidities [23]	STEP TEENS RCT: once-weekly semaglutide 2.4 mg produced a 16.1% reduction in BMI at 68 weeks vs. 0.6% placebo, with improvements in triglycerides, cholesterol, and glycaemic parameters. FDA approved for adolescent obesity in December 2022. Long-term cardiovascular outcome data in paediatric populations remain absent. Complements, not replaces, population-level prevention [23]
Digital and adolescent	Peer-led digital programmes; motivational interviewing; mental health integration; app-based self-monitoring [16]	Promising, highly engaging avenues requiring long-term RCT validation to confirm sustained BMI z-score reduction. Integrated approaches addressing mental health alongside physical health show particular promise [16]

The evidence that MHO carries substantially elevated long-term risk [13] strengthens the case for universal rather than selective preventive action. Consistent with DOHaD, optimal prevention begins prenatally [11]. The World Health Organization (WHO) antenatal care recommendations support gestational weight management and breastfeeding promotion, associated with 13-26% childhood obesity risk reduction, as globally applicable strategies [32]. School-based multicomponent programmes demonstrate significant BMI z-score reductions across 153 RCTs globally, with effectiveness enhanced by family involvement, sustained duration, and cultural tailoring [33]. Evidence from LMICs is increasing but remains limited [34]. Family-based behavioural interventions deliver clinically meaningful benefits when sustained over six months or longer [35]. Systematic review evidence across multiple countries confirms that sugar-sweetened beverage taxes reduce purchases and dietary sugar intake [36], with the UK Soft Drinks Industry Levy (SDIL) demonstrating an equity benefit in lower-income households [37]. Similar levies in Mexico, Chile, and South Africa confirm translatability across diverse economic settings. Whole community approaches that coordinate action across schools, primary care, food environments, and local government represent the most promising structural model for sustained population-level impact, though rigorous long-term outcome evidence across diverse settings remains limited [33,34]. The WHO Global Action Plan on Physical Activity 2018-2030 provides an internationally applicable structural framework [38]. Adolescent-specific interventions are most effective when peer-led, digitally delivered, and framed around adolescent values. Long-term RCT evidence confirms that sustained clinical benefit remains limited [16]. The STEP TEENS trial demonstrated that once-weekly semaglutide 2.4 mg produced a 16.1% BMI change at 68 weeks (vs. 0.6% placebo) with improvements in cardiometabolic risk factors in adolescents aged 12-17 with obesity [23]. However, long-term cardiovascular outcome data are absent, and treatment access is severely constrained in LMICs. Engagement with the commercial determinants of the food environment, including platform-level governance of algorithmic food marketing, is a necessary component of any serious policy response [29].

Synthesis and implications for policy and clinical practice

**Figure 2:**
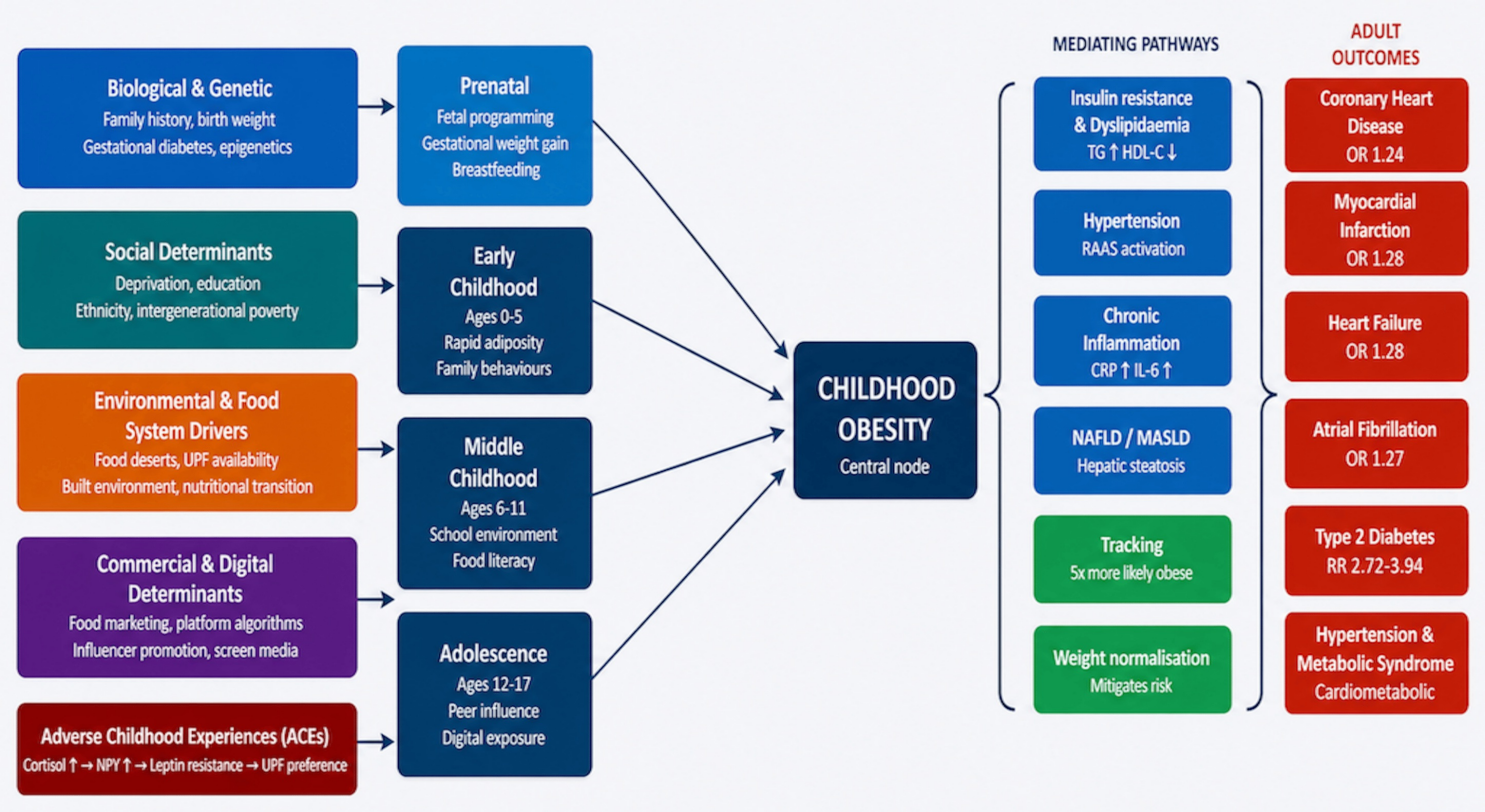
Life course conceptual framework linking biological, social, environmental, and commercial determinants of childhood obesity to adult cardiometabolic disease UPF: ultraprocessed food; NPY: neuropeptide Y; TG: triglycerides; HDL-C: high-density lipoprotein cholesterol; CRP: C-reactive protein; IL-6: interleukin 6; RAAS: renin-angiotensin-aldosterone system; NAFLD: non-alcoholic fatty liver disease; MASLD: metabolic dysfunction-associated steatotic liver disease Figure created by the authors using Google Slides (Google LLC, Mountain View, California, United States)

The evidence reviewed here converges on a clear conclusion: childhood obesity is causally linked to adult cardiometabolic disease, with Mendelian randomisation confirming associations with coronary heart disease, myocardial infarction, heart failure, and atrial fibrillation through specific mediating pathways [12]. The MHO finding, pooled RR 2.72 for adult diabetes compared with MHNW peers [13], contradicts the assumption that metabolic normality at assessment implies long-term safety and mandates universal early intervention. Combined with evidence from Juonala et al., this establishes weight normalisation before adulthood as the primary clinical goal, necessitating early, sustained, universal intervention [5]. In clinical practice, paediatric and primary care clinicians should apply systematic cardiometabolic screening to all children with significant obesity regardless of current metabolic status, including blood pressure, fasting lipid profile, fasting glucose, HbA1c, and alanine aminotransferase as a hepatic screen for MASLD [16]. Children with severe or early-onset obesity should be assessed for monogenic causes, including MC4R gene mutations, leptin receptor (LEPR) deficiency, and POMC deficiency, now treatable with targeted agents [16]. Standard Western BMI z-scores may underdiagnose visceral adiposity in South Asian and East Asian children, who exhibit higher cardiometabolic risk at lower BMI thresholds, and here waist-to-height ratio provides a complementary assessment [16]. Children of African ancestry may demonstrate different relationships between BMI, body composition, and cardiometabolic risk, highlighting the limitations of relying solely on BMI-based classifications to estimate the cardiometabolic risk [16]. Population-level improvements require equity-focused structural interventions and fiscal and regulatory measures with demonstrated equity benefits, together with governance, representing high-priority instruments globally. The life course conceptual framework linking upstream determinants to adult cardiometabolic outcomes is illustrated in Figure 2.

Limitations

As a narrative review, this synthesis is subject to selection bias, and the strength of conclusions varies across thematic sections. The evidence base is predominantly derived from high-income countries in North America and Northern Europe, with limited representation from South Asia, Africa, and Latin America. The Mendelian randomisation evidence is based primarily on European ancestry GWAS data, and it may not estimate the true causal hazards in ethnically diverse populations. Publication bias may additionally inflate apparent associations in the observational literature. BMI is an imperfect proxy for body fatness across age, sex, and ethnicity.

## Conclusions

Childhood obesity should be regarded not merely as a risk factor but as an early life manifestation of lifelong cardiometabolic vulnerability requiring intervention across the life course. The cumulative weight of genetic, longitudinal, and mechanistic evidence strongly supports universal, early, and sustained preventive action. Despite the strength of the available evidence, policy responses have not consistently matched the scale of the challenge, and this suggests that the principal barriers to progress now lie within structural, commercial, and policy domains rather than scientific uncertainty.

The coming years represent a critical window. A life course, whole systems public health approach addressing biological, social, commercial, environmental, and structural determinants simultaneously represents the most comprehensive and promising framework currently available. Fiscal and regulatory interventions with demonstrated equity benefits, governance of commercial food environments, and contextually adapted low-resource strategies for transitional economies should be considered as priority policy instruments rather than optional additions.

Future research should prioritise trans-ethnic causal studies, long-term cardiovascular outcome trials of emerging pharmacological agents in paediatric populations, the characterisation of MHO natural history across diverse global populations, the evaluation of structural policy interventions in LMICs, the establishment of longitudinal birth cohorts in South Asia and Africa, and the investigation of platform-level commercial food marketing governance as an upstream prevention strategy.
